# Chronic manganese exposure and neuroaffective disorder development in Parkinson’s disease models: Insights from oxidative stress measurements

**DOI:** 10.1192/j.eurpsy.2025.1328

**Published:** 2025-08-26

**Authors:** H. Harifi, H. Hami, L. Bikjdaouene

**Affiliations:** 1 Laboratory of Biology and Health, Faculty of Science, Ibn Tofail University, Kenitra, Morocco

## Abstract

**Introduction:**

Parkinson’s disease (PD) and manganism share similar etiological cellular mechanisms, notably the accumulation of manganese (Mn) in mitochondria, which triggers oxidative stress and neurotoxicity. Both disorders are characterized by irreversible motor and non-motor symptoms, including frequent depressive and anxiety disorders.

**Objectives:**

This study evaluates the levels of oxidative stress in various nervous structures affected by PD and examines the correlation between Mn exposure and the resulting neuroaffective disorders.

**Methods:**

Male Wistar rats were administered varying doses of Mn (6 mg/kg, 25 mg/kg, and 40 mg/kg) via daily intraperitoneal injections for 12 weeks. Body weights were monitored weekly. Following exposure, the rats were euthanized, and the striatum, frontal cortex, prefrontal cortex, hippocampus, and olfactory bulb were harvested for analysis. Tests were conducted to measure levels of nitric oxide (NO), lipid peroxidation (LPO), and catalase activity (CAT), indicators of oxidative stress.

**Results:**

Chronic exposure to Mn at doses of 25 and 40 mg/kg significantly induced PD-like symptoms and altered affective behavior, leading to anxiety and depressive behaviors in rats. Notably, these doses resulted in increased LPO levels in the hippocampus, frontal cortex, and prefrontal cortex. Conversely, the 40 mg/kg dose caused an increase in LPO across all tested structures. Elevated doses also resulted in increased NO levels and CAT activity across all structures, contrasting with the 6 mg/kg dose, which did not significantly differ from control values.

**Conclusions:**

The findings demonstrate that chronic Mn exposure has neurotoxic effects that adversely affect neuroaffective behavior, manifesting as symptoms of depression and anxiety. These results highlight the critical association between Mn-induced oxidative stress and the development of neuroaffective disorders.

**Disclosure of Interest:**

None Declared

